# Brain-hemispheric differences in the premotor area for motor planning: An approach based on corticomuscular connectivity during motor decision-making

**DOI:** 10.1016/j.neuroimage.2025.121230

**Published:** 2025-05-15

**Authors:** Leonardo A. Cano, Ana L. Albarracín, Fernando D. Farfán, Eduardo Fernández

**Affiliations:** a Neuroscience and Applied Technologies Laboratory (LINTEC), Instituto Superior de Investigaciones Biológicas (INSIBIO), National Scientific and Technical Research Council (CONICET), and Bioengineering Department, Faculty of Exact Sciences and Technology (FACET), National University of Tucuman (UNT), San Miguel de Tucumán 4000, Argentina; b Faculty of Physical Education (FACDEF), National University of Tucuman (UNT), San Miguel de Tucumán 4000, Argentina; c Institute of Bioengineering, Miguel Hernandez University (UMH), Elche 03202, Spain; d Research Networking Center in Bioengineering, Biomaterials and Nanomedicine (CIBER-BBN), Madrid 28029, Spain

**Keywords:** Motor planning, Reaction time, Corticomuscular coherence, Decision-making, Premotor area, EEG-EMG

## Abstract

This study investigates the role of the premotor area (PMA) in motor planning during decision-making, focusing on differences between brain hemispheres. A cross-sectional assessment was conducted involving seventeen right-handed participants who performed tasks requiring responses with either hand to visual stimuli. Motion capture, EEG and EMG signals were collected to analyze corticomuscular coherence (CMC) in the beta and gamma bands across four motor-related cortical areas. Findings revealed significant beta-band CMC between anterior deltoids and contralateral PMA before stimulus onset in simple reaction tasks. Moreover, significant beta-band CMC was observed between the left anterior deltoid and the right PMA during the motor planning phase, prior to the onset of muscle contraction, corresponding with shorter planning times. This connectivity pattern was consistent across both simple and complex reaction tasks, indicating that the PMA plays a crucial role during decision-making. Notably, motor planning for the right hand did not exhibit the same connectivity pattern, suggesting more complex cognitive processes. These results emphasize the distinct functional roles of the left and right hemispheres in motor planning and underscore the importance of CMC in understanding the neural mechanisms underlying motor control. This study contributes to the theoretical framework of motor decision-making and offers insights for future research on motor planning and rehabilitation strategies.

## Highlights

• Motor planning of the hand movements are not symmetrical across brain hemispheres.
• The premotor area plays a critical role in motor decision-making.
• A novel approach to exploring motor planning through functional connectivity within very short time intervals.

## 1 Introduction

In the field of motor control research, motor planning has been extensively studied in close relation to decision-making. Schmidt and Lee (2020) proposed a conceptual model based on information processing theory, which encompasses the perception-decision-response process. In short, perception is the phase in which individuals receive both internal and external information. During this phase, a selective process occurs, filtering out irrelevant information while utilizing the relevant data to determine the parameters for responding (or not) to a given stimulus. The time interval between stimulus onset and the initiation of a motor response is operationally defined as Reaction Time (RT), a measure widely used in the literature (Badau et al., 2018; Chiu et al., 2017; Dexheimer et al., 2022; Nuri et al., 2013; Vaeyens et al., 2007). During RT, several central and peripheral processes are involved in generating a response. These include scanning the environment to identify the object of interest, deciding on the response and its features, and evoking the motor program that initiates the planned action. This set of processes can collectively be defined as decision-making.

According to Wong et al. (2015), perceptual decision-making is primarily concerned with *what to do*, while motor planning (i.e., motor decision-making) addresses *how to do* it. Perceptual decision-making involves evaluating options and selecting a motor goal. In contrast, motor planning involves devising the operational steps required to execute the chosen action, involving sensorimotor integration and preparation within motor-related brain regions. This intricate process primarily engages the sensorimotor areas of the cerebral cortex (Brown and Cooke, 1981; Kakei et al., 2001, 1999; Kilavik et al., 2013; Scott and Kalaska, 1997) as well as subcortical structures such as the cerebellum, basal ganglia, thalamus, among others (Kandel et al., 1997; Reis et al., 2019; Tsang et al., 2012). Previous studies (Cisek and Kalaska, 2005; Dum and Strick, 1991; Fine and Hayden, 2022; Graziano and Aflalo, 2007; He et al., 1995; Lemon, 2008; Neige et al., 2018) suggested that certain aspects of motor planning involves the premotor area (PMA) and supplementary motor area. These regions play a crucial role in coordinating and orchestrating motor programs and actions. Synchronous brain rhythms act as a dynamic mechanism for organizing neural activity across large-scale neuronal networks and precisely regulating the timing of neuronal firing. The neural oscillations from motor-related areas are effectively propagated along the corticospinal tract, supporting the seamless integration of central and peripheral motor functions (Yang et al., 2016). The cortex generates commands that travel through the spinal cord to facilitate muscle activation and control limb movements. In this line, the integration between motor-related areas and muscles has been extensively studied over the past three decades in both humans (Conway et al., 1995) and monkeys (Baker et al., 1997), using non-invasive techniques such as electromyography (EMG) and electroencephalography (EEG). Electrophysiological signals (e.g., EEG, EMG) provide high-temporal resolution data, enabling neural activity to be analyzed in short time intervals. The Corticomuscular Coherence (CMC) has been employed to assess the coupling between EEG and EMG signals.

Over the past decade, CMC has become a key tool in motor control research (Boonstra, 2013; Liu et al., 2019). Since then, an increasing number of studies have probed the details of this connection, both in physiological and pathological conditions. In this line, there is still divergence in the findings reported and a consensus regarding the specific frequency bands associated with motor control. A pioneer study in humans (Conway et al., 1995) identified a coupling in the beta band. Posterior observations differentiated the production of static force (beta-band CMC) from dynamic force (gamma-band CMC) (Gwin and Ferris, 2012; Omlor et al., 2007). Consistently, higher amplitude in beta-band CMC was associated with better motor control performance (Desmyttere et al., 2018; Kristeva et al., 2007; Reyes et al., 2017), force production stability and accuracy (Elie et al., 2021), and modulation by visual feedback during motor tasks (L’Abbate et al., 2022). Also, aging was associated with a reduction of beta-band CMC during sustained contractions of upper limb muscles (Bayram et al., 2015; Graziadio et al., 2010). Moreover, beta-band CMC have been used for characterizing the agonist/antagonist relationship. Dal Maso et al. (2017) observed that beta-band CMC magnitude decreased more significantly in antagonist muscles than in agonist muscles as torque levels increased during static knee contractions in both strength- and endurance-trained individuals. In post-stroke patients, Braun et al. (2007) reported that CMC predominantly occurs within the 10–23 Hz frequency range. In line with this, Fauvet et al. (2021) showed an increase in beta-band CMC in antagonist muscles of post-stroke patients compared to controls during the acceleration phase of elbow extension movements.

Studies utilizing CMC (Kenville et al., 2020; Tun et al., 2021; Yang et al., 2016) have shown that the directionality of motor commands from the cortex to muscles is modulated in the beta band. Mehrkanoon et al. (2014) observed that beta-band CMC is prominent during sustained contractions but diminishes prior to movement onset, replaced by transient synchronization in the alpha and gamma bands during dynamic force outputs. These dynamic shifts reflect bidirectional corticospinal interactions. Despite these advances, a comprehensive understanding of the central processes underpinning motor planning remains elusive. Recently, Bigot et al. (2011) introduced an alternative CMC computation method that leverages time-frequency analysis via continuous wavelet transform. This approach enables the analysis of data from large- and multi-joint movements (Dal Maso et al., 2017; Delcamp et al., 2023; Desmyttere et al., 2018; Elie et al., 2021; Fauvet et al., 2021; Glories et al., 2021; Tisseyre et al., 2022), offering insights beyond static motor tasks where decision-making could play a limited role. Since Pfurtscheller and Lopes da Silva (1999), along with subsequent studies, observed variations in beta oscillations before and after motor-related neurophysiological processes (commonly associated with the synchronized activity of neurons in specific regions of the motor cortex), CMC emerges as a logical approach to characterize cortex-to-muscle communication, even before the motor response becomes evident. While CMC analysis focused on periods of overt muscle contraction, where clear EMG signals allow for a direct examination of coupling between cortical and muscular activity, recent research (Zheng et al., 2025) has demonstrated that CMC can also be detected at the level of individual motor units. As EMG signals are essentially the summation of motor unit action potentials (MUAPs), they only become detectable once a sufficient number of MUAPs have been recruited. However, corticomuscular communication is established much earlier, detectable through specific motor units before the EMG signal becomes apparent. This underscores the importance of examining CMC at earlier stages of motor action. To capture this early activity, we employed a time-frequency analysis method based on the wavelet transform (Bigot et al., 2011), which allowed us to detect coupling that precede muscle contraction.

To our knowledge, this study is the first in computing CMC to analyze motor planning during situations that require decision-making. The aim of the present work was to address this lack by examining the functional connectivity between cortex and muscles during motor planning. In order to explore this mechanism, we designed an experimental protocol based on Go/no-go tasks. This protocol required the participants to make a decision regarding which hand to move to the target once the visual stimulus was presented. We hypothesized that motor planning process may modulate the beta-band CMC in the premotor area. To test this hypothesis, we compared beta- and gamma-band CMC across four motor-related cortical areas. In addition, we also explored whether pre-stimulus functional connectivity states vary across different levels of task complexity. This study could have significant implications for future research on exploring how the brain encodes motor planning during time intervals shorter than a half second.

## 2 Methods

### 2.1 Study design

This study is a cross-sectional assessment performed at Bioengineering Institute of Miguel Hernandez University of Elche, Spain. The procedure took place in a quiet room designed for neuroscience research, where the room temperature was maintained within the range of 20 to 24 °C. The room was equipped with amenities for noise isolation and minimizing distractions. For the estimation of the sample size required, an a priori power analysis was carried out with the G*Power v.3.1 software. For repeated measures ANOVA within factors, the software indicated a sample size of 16 subjects with an effect size = 0.30, α error = 0.05, and power = 0.80. The study was conducted by following the ethical guidelines established in the Declaration of Helsinki and approved by the ethics committee of the Miguel Hernandez University from Elche, Spain (Reference: IB.EFJ.04.21). All participants were previously instructed about the tasks and gave written informed consent.

### 2.2 Participants

Seventeen healthy volunteers (9 males, 8 females, age 28.66 ± 8.8 years) took part in a motor reaction study involving visual stimuli. A non-probabilistic sampling of volunteers was conducted among university community, reflecting a diverse pool of participants eager to contribute to the study. All participants self-reported as right-handed, with normal or corrected-to-normal vision, and no history of neurological or locomotor disorders. They confirmed that they did not have color blindness, which could influence their ability to distinguish stimuli. The inclusion of both male and female participants was a given, and the research design intentionally sought to reflect this diversity to ensure the generalizability of our findings. All participants were instructed to arrive at the laboratory in a rested state, avoiding any strenuous activity in the previous 24 h. The participants were asked to avoid any food or drink containing caffeine for at least 12 h preceding the session.

### 2.3 Experimental protocol

**Fig. 1:**
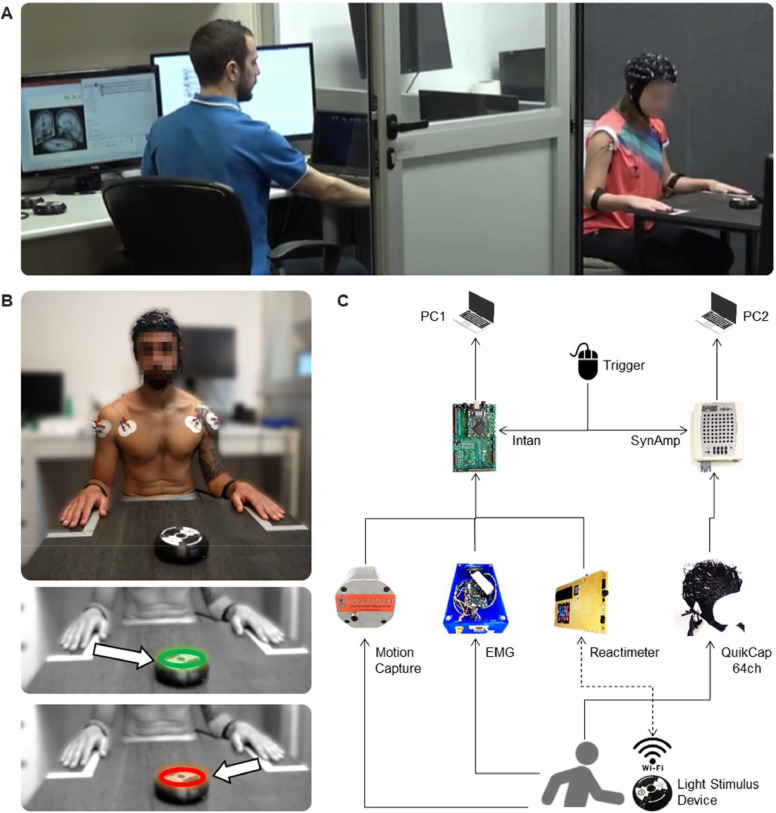
Experimental setup. (A) Photograph of the actual room prepared with experimental setup (B) Photograph depicting the initial position of the participant. The subfigures below illustrate the instructions given to the participant. When the green light turns on, the participant was instructed to move their right hand towards the device; instead, the red light indicated movement of the left hand. (C) Schematic diagram of the instrumentation and connections. Data acquisition was synchronized using an external pulse (trigger) and recorded by two systems (Intan and SynAmp). The acquired data was stored in separate computer files for further offline processing.

The experimental protocol was based on previous studies (Cano et al., 2024a, 2024b). The experiment consisted of sitting the participant in front of a table with their hands located in a predefined position (Fig 1A). A device (reactimeter) was placed on the table and in front of the participant. The reactimeter emits programmed lights following a predefined protocol. The device emits one-color light (red or green) and includes a motion sensor. When the participant places their hand on the reactimeter, the light turns off; then, it is possible to measure the time lapse between the light being turned on and turned off. The participant was instructed to associate the green color stimulus with right-hand movement, and the red color with left-hand movement (Fig 1B). Before the formal data collecting, a test trial was conducted to ensure the participant's understanding of the instructions. Two experimental conditions were established for motor tasks, and the sequence of the two conditions was consistent across all participants: (1) Simple Reaction (SR) condition: The device emitted only one predetermined color, so the participant knew in advance that the hand should be moved before the light appeared. The initial light color presentation was randomized, with half of the participants starting with the green light and the other half with the red light. Each participant performed one series of twenty repetitions with each hand. (2) Complex Reaction (CR) condition: The device randomly emitted green or red light, so the participant had to make a decision before moving any hand. One series of twenty repetitions was performed in this condition. Thus, each participant completed three series in total.

For both conditions, the minimum time between repetitions had to be 6 s; after that, the stimulus was spontaneously delivered. Participants were unaware of this information and were warned to remain attentive. The time for resting between series was 2 min. This permitted participants to rest from the sustained attentional demand (approximately 2 min) of each task and minimized mental dispersion from potential distractions. Consequently, participants’ attention on the entire experiment did not exceed 15 min in total. In order to avoid extra movements but the hands that could affect EEG recordings, the device was located 30 cm from the hands’ initial position. Additionally, participants were instructed to keep their gaze fixed on the device. The absence of a learning effect related to the repetitive motor task was verified. Supporting data and statistical analysis about the absence of learning effects can be found in the Supplementary Material in previous reports (Cano et al., 2024b).

### 2.4 Instruments

**Fig. 2:**
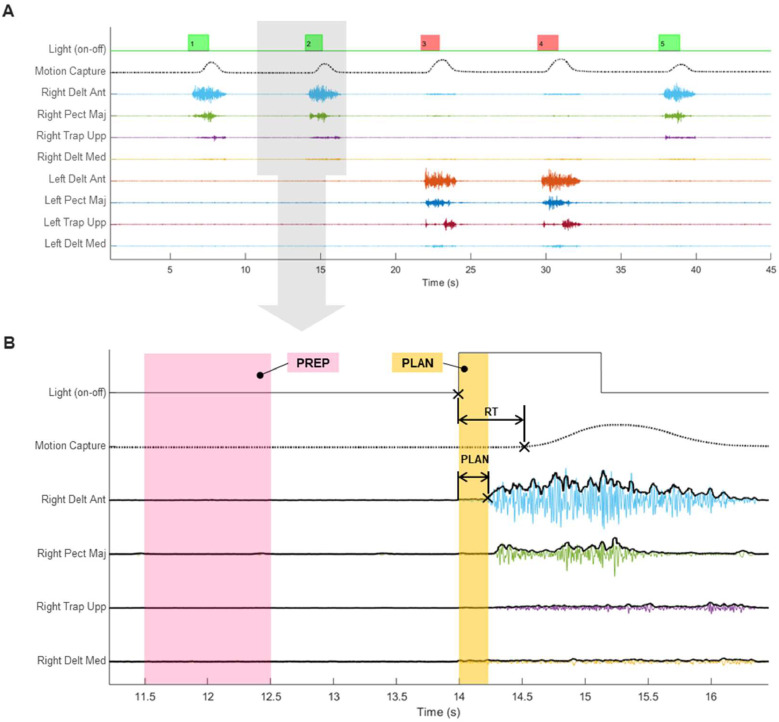
Diagram showing all synchronized time-series without EEG. (A) Five repetitions of complex reaction task over time. The upper line shows the reactimeter signal, binary on–off data are marked by green and red, depending on the light stimulus shown. The second line shows motion capture when either hand was moved. These data are an analog time-series scaled in millimeters derived from digital signals collected with a linear position transducer. The movement of either hand is detected when there is a change in the steady state, transitioning from a flat line to an increasing amplitude following the presentation of a visual stimulus. The next lines depict the normalized EMG data from eight muscles. (B) Zoom-in of the light gray rectangle in superior panel. The crosses (x) indicate the onset of light, movement, and muscle contraction. Preparation phase (PREP) is a 1-second time-window, starting at 2.5 s before the light turn-on (in pink). Reaction time (RT) is the interval between light turn-on and movement onset. Planning phase (PLAN) is the interval delimited between light turn-on and effective muscle contraction detected (in orange). The bold black line over the EMG signals is the envelope detected using the root mean square method.

We used the SynAmps RT system, 1 kHz sampling rate, and a 64-channel Quik-Cap helmet (Compumedics Neuroscan, Charlotte, NC, USA) for cortical activity recordings. EEG data were acquired with software Curry v7. Conductive gel was applied to the helmet electrodes, ensuring an impedance <25 kΩ. Superficial EMG recordings were acquired with RHA2000-series (Intan, Los Angeles, CA, USA) acquisition system with 16-channel amplifier and 25 kHz sampling rate. Muscle activity data were collected using Ag/AgCl button electrodes (Dormo, Barcelona, Spain); skin surface preparation and electrode placements were performed following SENIAM guidelines. To measure the time, we used a system designed and manufactured in our laboratory (LINTEC, San Miguel de Tucuman, Argentina). It consists of a control center programmed for sending the signal via WiFi to the device to turn on/off the light. The reactimeter was developed to fulfill specific requirements of the protocol that were not supported by commercial systems. For example, it incorporates an external synchronization system with other devices, enabling real-time data acquisition without delay, and offers versatility in programming stimulus delivery (number of devices, colors, timing, and pauses). A linear position transducer with 1 kHz sampling rate (WinLaborat, Buenos Aires, Argentina) was attached to both forearms by adjustable tapes in order to detect hands movement. The data from the position transducer and the reactimeter were acquired using the Intan board’s auxiliary digital channels. Cables connected from both instruments to the Intan facilitated this acquisition. The digital channels recorded data from these two instruments as time series, operating in parallel with the EMG recording. This synchronization ensured continuous alignment of all three types of signals, thereby preventing any lag or delay that could compromise data accuracy. An external synchronization system (trigger) was activated by the operator before the test began. The trigger was configured to simultaneously send a signal to both the Intan and SynAmps systems (Fig 1C). Both recordings were acquired separately, but synchronized later by signal pre-processing (Fig 2A).

### 2.5 Agonist muscle identification

Data from eight muscles (four per side) were collected: *Trapezius (upper fibers), Deltoideus Anterior, Deltoideus Medius, and Pectoralis Major (pars clavicularis)*. To determine muscle participation, we relied on the triphasic EMG pattern (Cheron et al., 2007; Forgaard et al., 2013). As Correia et al. (2022) explained, this pattern consists of three sequential bursts of EMG activity interspersed by periods of EMG silence, and it is mostly observed in fast movements. The first burst corresponds to the agonist that initiates the planned movement. Then, there is an increase in antagonist muscle activity to provide a braking force when approaching the target. Finally, another smaller agonist burst fine-tunes the final movement position. Among the four muscles in each arm, the *Deltoideus Anterior* (AD) was identified as the agonist muscle in every condition. This identification was carried out by following the established methodology outlined in prior work (Teruya et al., 2021). This procedure consisted in normalizing the EMG signal with the interval max value within the reaction time, for each separated repetition. Second, detecting the envelopes with the root mean square (RMS) of every repetition. Third, the onset of the effective contraction was determined as the envelope amplitude exceeding three standard deviations over the basal state. The same criteria were applied for determining hand movement onset (see the crosses × in Fig. 2B), corresponding to the end-point of reaction time.

### 2.6 Signal pre-processing

The analysis was performed offline with Matlab (MathWorks, Natick, MA, USA), version 2020b The motion data from the position transducer was analyzed using classical kinematics formulations for obtaining displacement data. The reactimeter data were kept as binary on–off data. EMG recordings were resampled at 1 kHz. We applied a zero-lag fifth-order Butterworth filter (band-pass 10–100 Hz, band-stop 49–51 Hz). As we have mention above, AD was identified as the agonist muscle. Determining the agonist muscle onset is crucial to separate planning phase for the subsequent procedures. We used the unrectified and non-normalized EMG signal to meet the theoretical demands and uphold the practical justifications for the computation of CMC (Bigot et al., 2011). EEG signals were pre-processed in two stages. In the first stage, the EEGLAB toolbox (Delorme and Makeig, 2004) was used. The channels were re-referenced to the average, and high-pass 4 Hz filtered. Next, a visual inspection using the ‘scroll data’ and ‘channel properties’ functions corroborated no big artifacts coming from non-typical biological sources. As Klug and Gramann (2021) recommended, we avoided eliminating channels or segments of the recording that contain artifacts of apparent biological origin (muscle contractions, eye-blinking, eye movement) before Independent Component Analysis (ICA). The next step was to perform ICA to identify signal components that could be covering the cortical signal. The RunICA algorithm was applied on 64 channels. Next, the ICLabel algorithm was applied to classify Independent Components (ICs). ICs identified as ‘muscle’, ‘eye’, ‘bad channel’, and ‘heart’ with a probability higher than 0.6 were labeled. After a visual inspection of each IC, some of them were discarded. We discarded 19.97 ± 5.72 from 64 ICs per series. Finally, the signal was reconstructed without the discarded components. The second stage was performed in Matlab, and a zero-lag fifth-order Butterworth filter (band-pass 10–100 Hz) was applied.

### 2.7 Phases identification and data segmentation

Specific events during the task (i.e., light turn-on, muscle contraction onset) were used to determine the onset and finalization of the phases of interest: preparation (PREP) and planning (PLAN), highlighted in Fig. 2B PREP corresponds to the period during which the participant awaited the presentation of the visual stimulus. Analyzing this phase allows for the evaluation of connectivity states during an interval sufficiently distant from any stimulus or motor action. This makes it possible to characterize the brain's preparatory state in conditions of certainty (simple reaction) or uncertainty (complex reaction). For subsequent analysis, a fixed 1-second window was selected to represent PREP, specifically between 2.5 and 1.5 s before the light turned on for each repetition. The choice of a 1-second window ensures sufficient duration to capture potential connectivity states, if present. Additionally, positioning the window at 2.5 s prior to stimulus onset minimizes the likelihood of anticipatory movements influencing the CMC computations. PLAN corresponds to the interval between light turning on and the onset of muscle contraction, representing the duration of the motor planning process. PLAN represents the time interval when the participant makes a decision about which hand should move and to plan the motor action. The time series of EMG and EEG data were extracted within the temporal segments identified for the PREP and PLAN phases for each repetition. These segmented time series were subsequently used for further analyses. An offline inspection looking at all data was conducted to detect execution errors. The criterion for determining which repetitions should be discarded was based on the hand movement data captured by the position transducer; for example, if movement was detected before the stimulus appeared, or if there was incorrect moved-hand relative to the presented color, or if there was simultaneous movement of both hands. Repetitions with errors were excluded from the analysis. In the SR condition, 1.3 % of the repetitions for the right hand and 0.3 % for the left hand were discarded. In the CR condition, 3 % and 3.1 % were discarded for the right and left hand, respectively. After error removal, an average of 19.76 ± 0.35 repetitions were analyzed for the SR condition, and 19.35 ± 0.78 repetitions were analyzed for the CR condition.

### 2.8 Corticomuscular coherence (CMC) computation

The procedure to compute CMC followed the method presented by Bigot et al. (2011), and following recommendations of recent implementations (Dal Maso et al., 2017; Delcamp et al., 2023; Desmyttere et al., 2018; Elie et al., 2021; Fauvet et al., 2021; Glories et al., 2021). This procedure can be summarized as the following steps: (1) select two time series (e.g., EEG-EMG); (2) select the segment to analyze; (3) apply the continuous wavelet transform of both segmented signals; (4) compute the ‘Cross-Spectrum’ between each signal pair, and compute the ‘Mean Auto-Spectrum’ for both signals separately; (5) the ‘Magnitude-Squared Coherence’, that is the ‘Mean Cross-Spectrum’ normalized by the ‘Mean Auto-Spectrum’ of each signal, was calculated in the time-frequency domain, and only the values of the ‘Magnitude-Squared Coherence’ where the ‘Mean Cross-Spectrum’ was statistically significant were retained; (6) compute the CMC as the single value (mean) within each time-frequency window of interest. Detailed explanations with a step-by-step illustrated example (Fig S1) can be seen in the Supplementary Material.

**Fig. 3:**
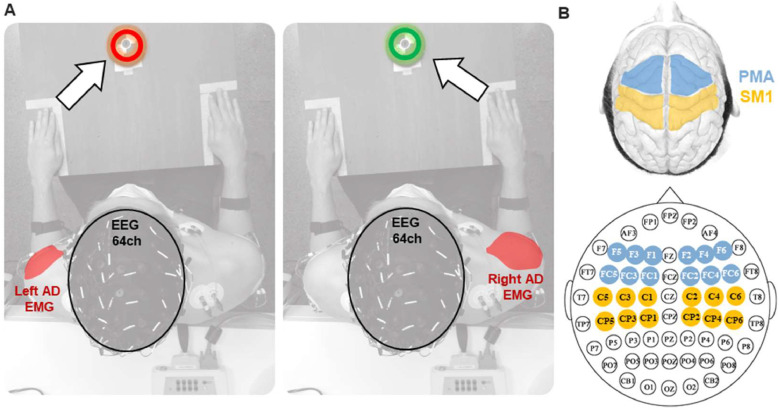
Top view of the experimental setup and EEG-sensors distribution. (A) The left panel depicts when the red light turn-on, the left Anterior Deltoid (AD) is the agonist muscle. The right panel depicts the opposite, when the green light turn-on, the right AD is the agonist muscle. Sixty-four values of CMC were computed for each condition. (B) The upper panel depicts a top view of a brain cortex model. The Premotor area (PMA) is highlighted in light blue for both hemispheres, and the Primary Sensorimotor area (SM1) in yellow. The lower panel depicts EEG configuration for 64 channels according to the 10–10 international system. The electrodes corresponding to PMA and SM1 are colored with the same color code.

This procedure was performed using the EMG signal from the AD muscle of the corresponding limb, and the 64 EEG-channel (Fig. 3A). To compute CMC during PREP, we used a fixed-time window delimited as described in Section 2.6. To compute CMC during the PLAN phase, whose duration varies across trials, we applied a time normalization procedure described by Fauvet et al. (2019). This normalization step was designed as to preserve frequency content of signals and enable point-wise comparison between segments of different durations. As a result, time-frequency maps in Figure S1 are expressed as a percentage of the normalized time within the PLAN phase, rather than absolute time, where 0 % corresponds to the stimulus onset and 100 % to muscle contraction onset. The frequency limits for the window of interest were established within beta band (15 to 30 Hz), and within gamma band (30 to 45 Hz). Thus, we obtained a single value of CMC magnitude for every EEG-channel with corresponding AD muscle, for each condition. The decision to focus on the 30–45 Hz range for the gamma band was based on previous studies evaluating CMC that specifically use this range for gamma (Gwin and Ferris, 2012; Mehrkanoon et al., 2014; Omlor et al., 2007; Tun et al., 2021), also referred as ‘low-gamma’ (Kenville et al., 2020).

### 2.9 Regions of interest

Four motor-related cortical regions of interest (ROIs) were studied. The selection of EEG electrodes to represent ROIs was consistent among participants based on 10–10 international system of distribution. For the premotor area (PMA), the following channels were pooled: F1, F3, F5, FC1, FC3, FC5 (left hemisphere) and F2, F4, F6, FC2, FC4, FC6 (right hemisphere). For the primary sensorimotor area (SM1), channels C1, C3, C5, CP1, CP3, CP5 (left hemisphere) and C2, C4, C6, CP2, CP4, CP6 (right hemisphere) (Fig. 3B). Therefore, we averaged the CMC values from the pool of channels representing the functional connectivity of each ROI to obtain a single representative value. By averaging the data from six electrodes per ROI, rather than using the most representative, the connectivity can be effectively analyzed across a wide range of electrode coverage across participants, thereby improving the reliability of the results. In addition, for maximum resolution of topographic visualization over the scalp, we used the EEGLAB 'topoplot' function to plot the normalized CMC values of each of the 64 channels and the agonist muscle.

### 2.10 Statistical analysis

Statistical analysis was performed using Matlab 2020b The data were checked for normality using the Shapiro-Wilk test (α = 0.05). Firstly, we performed an analysis of planning time. Normal distribution of data was verified. We calculated the means and standard deviations for each participant separately, for each hand and for each condition. Then, the input data for the following statistical analyses were the previously calculated means. To assess the differences in planning time across conditions and hands, we performed a two-way repeated measures ANOVA (conditions and hands) followed by Bonferroni post-hoc analysis. The analysis included both main effects and their interaction. Type I error rate was set at the conventional significance level (α = 0.05). Secondly, we performed an analysis of CMC magnitude among ROIs across conditions, for both beta and gamma band. The normal distribution was not confirmed (*p* < 0.05), requiring the use of non-parametric tests for subsequent analyses. We calculated the median, minimum, and maximum for the group of participants for each ROI, for each hand and for each condition. The Friedman test was chosen to evaluate the overall differences between the ROIs, for each hand and for each condition. The Friedman test is a non-parametric alternative to the repeated measures ANOVA and is used to detect differences across multiple test. Upon obtaining significant results from the Friedman test, we proceeded to conduct pairwise comparisons using the Nemenyi test. This method is suitable for post-hoc analysis following a significant Friedman test and allows for the identification of specific group differences. Each pairwise comparison was adjusted for multiple comparisons to control the family-wise error rate. The magnitude of difference between comparisons was calculated using Kendall’s W, similar to effect size, as small (<0.2), medium (0.2–0.5), and large (>0.5). Type I error rate was set at the conventional significance level (α = 0.05).

## 3 Results

### 3.1 Analysis of planning time

The two-way repeated measures ANOVA indicated a significant main effect of condition (F (1, 16) = 134.47, *p* < 0.001, η_p_^2^ = 0.81), indicating a difference in planning time between simple and complex reaction. Similarly, significant main effect of hand (F (1, 16) = 11.25, *p* = 0.002, η_p_^2^ = 0.26), indicating a difference in planning time between dominant (right) and non-dominant (left) hands. The interaction between condition and hand was not statistically significant (F (1, 16) = 0.94, *p* = 0.339, η_p_^2^ = 0.03), suggesting no evidence that the combined effect of condition and hand produces a different impact on planning times. In other words, the planning times for the right and left hands were consistent within each condition. The post-hoc paired *t*-test using the Bonferroni correction (α = 0.0083) indicated that the means of the every pairs were significantly different between them. These differences indicate that the increasing task complexity was reflected in planning times, in line with expectation. Furthermore, these results revealed that planning times for the left hand was significantly shorter than the right hand, consistently for both conditions. Table 1 shows a summary of the participants’ performance for each hand in each condition. For the subsequent analyses on CMC computation, we used only repetitions in the range of the mean ± 2 standard deviations for each participant and each condition. We decided to select this range of repetitions –around 95 %– to exclude outliers reducing intra-subject variability.

**Table 1:** Planning times in milliseconds, expressed as mean ± standard deviation for each participant.

Participants	Planning time (ms)			
	Simple Reaction (SR)		Complex Reaction (CR)	
	Right hand (dominant)	Left hand (non-dominant)	Right hand (dominant)	Left hand (non-dominant)
P1	218 ± 90	151 ± 109	322 ± 123	255 ± 128
P2	230 ± 110	144 ± 52	225 ± 95	209 ± 87
P4	164 ± 52	156 ± 43	245 ± 45	182 ± 86
P5	149 ± 72	141 ± 62	238 ± 91	193 ± 88
P6	215 ± 69	137 ± 57	347 ± 72	284 ± 139
P7	197 ± 60	153 ± 97	302 ± 164	248 ± 129
P8	145 ± 53	111 ± 55	277 ± 97	174 ± 112
P9	182 ± 66	139 ± 88	267 ± 98	266 ± 109
P10	165 ± 81	207 ± 57	211 ± 94	228 ± 112
P11	146 ± 43	157 ± 34	238 ± 114	162 ± 90
P13	188 ± 61	162 ± 86	334 ± 61	271 ± 111
P14	181 ± 78	130 ± 92	316 ± 151	286 ± 141
P15	224 ± 62	193 ± 51	286 ± 140	248 ± 146
P16	215 ± 108	197 ± 122	306 ± 68	266 ± 129
P17	138 ± 54	120 ± 47	243 ± 70	242 ± 104
P18	156 ± 50	187 ± 51	258 ± 78	247 ± 89
P19	215 ± 85	207 ± 115	255 ± 121	235 ± 127
**Group Average**	**184 ± 31**	**158 ± 30**	**275 ± 41**	**235 ± 38**

### 3.2 Analysis of corticomuscular coherence (CMC) during preparation (PREP) phase

Table 2 presents the results for the beta-band CMC in each region of interest (ROI) during PREP. Firstly, for Simple Reaction (SR) condition, we tested CMC for the Right Anterior Deltoid. The Friedman test indicated a significant (χ² = 26.85, *p* < 0.0001) and large effect (*W* = 0.53). Pairwise comparisons revealed that the CMC in left PMA was significantly greater than right PMA (*Q* = 5.44, *p* < 0.001), left SM1 (*Q* = 3.75, *p* < 0.05), and right SM1 (*Q* = 6.95, *p* < 0.001). Secondly, we tested CMC for the Left Anterior Deltoid. The Friedman test indicated a significant effect (χ² = 17.54, *p* < 0.0001) and medium effect (*W* = 0.34). Pairwise comparisons revealed that the CMC in right PMA was significantly greater than left PMA (*Q* = 3.75, *p* < 0.05), left SM1 (*Q* = 5.45, *p* < 0.001), and right SM1 (*Q* = 4.69, *p* < 0.005). For Complex Reaction (CR) condition, the Friedman test did not show significant effects. Consequently, no further pairwise comparisons were necessary for these variables.

**Table 2:** Beta-band Corticomuscular coherence (CMC) during preparation phase, expressed as the median values [min – max] in arbitrary units (a.u.).

Condition	Hand to move	Left PMA (a.u.)	Right PMA (a.u.)	Left SM1 (a.u.)	Right SM1 (a.u.)	Effect size (Kendall’s W)
Simple Reaction (SR)	Right hand	0.047* [0.019 - 0.089]	0.035 [0.003 - 0.077]	0.033 [0.015 - 0.067]	0.031 [0.005 - 0.076]	0.53
	Left hand	0.042 [0.014 - 0.055]	0.048 * [0.023 - 0.081]	0.037 [0.019 - 0.063]	0.035 [0.021 - 0.053]	0.34
Complex Reaction (CR)	Right hand	0.08 [0.057 - 0.124]	0.08 [0.052 - 0.111]	0.078 [0.039 - 0.123]	0.079 [0.039 - 0.126]	NS
	Left hand	0.069 [0.02 - 0.117]	0.078 [0.025 - 0.107]	0.083 [0.01 - 0.101]	0.073 [0.019 - 0.135]	NS

⁎ *p* < 0.0001, PMA = Premotor area, SM1 = Primary Sensorimotor area, NS = No significant.

Table 3 presents the results for the gamma-band CMC in each ROI during PREP. For the both conditions, SR and CR, we tested CMC for the Right and Left Anterior Deltoids. The Friedman test did not show significant effects in any case. Consequently, no further pairwise comparisons were necessary for these variables.

**Table 3:** Gamma-band Corticomuscular coherence (CMC) during preparation phase, expressed as the median values [min – max] in arbitrary units (a.u.).

Condition	Hand to move	Left PMA (a.u.)	Right PMA (a.u.)	Left SM1 (a.u.)	Right SM1 (a.u.)	Effect size (Kendall’s W)
Simple Reaction (SR)	Right hand	0.011 [0.001 - 0.036]	0.008 [0.001 - 0.037]	0.006 [0.001 - 0.028]	0.005 [0.001 - 0.034]	NS
	Left hand	0.004 [0.001 - 0.021]	0.005 [0.001 - 0.024]	0.004 [0.001 - 0.021]	0.004 [0.001 - 0.015]	NS
Complex Reaction (CR)	Right hand	0.027 [0.005 - 0.091]	0.029 [0.003 - 0.086]	0.018 [0.001 - 0.079]	0.019 [0.001 - 0.076]	NS
	Left hand	0.018 [0.002 - 0.057]	0.016 [0.006 - 0.04]	0.015 [0.001 - 0.038]	0.016 [0.001 - 0.03]	NS

PMA = Premotor area, SM1 = Primary Sensorimotor area, NS = No significant.

**Fig. 4:**
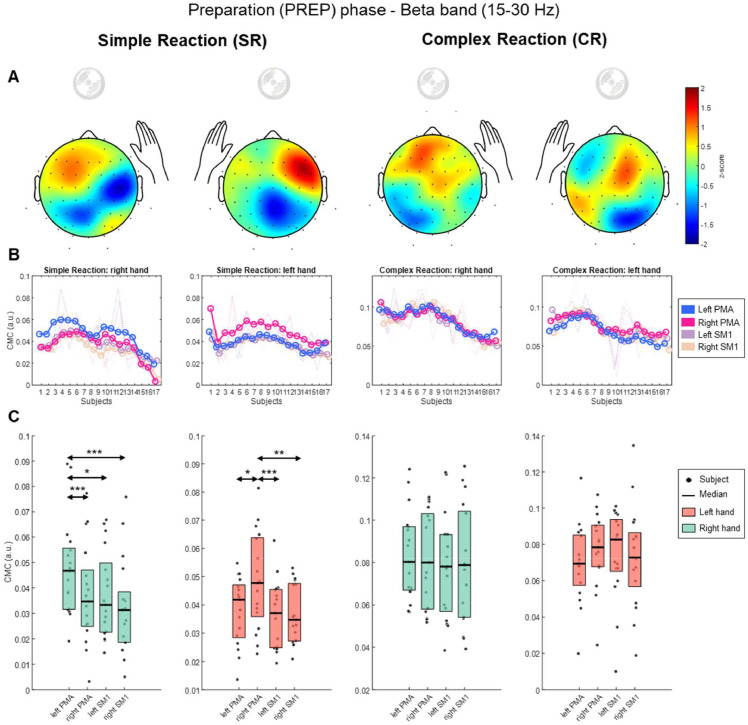
Beta-band Corticomuscular coherence (CMC) during preparation (PREP) phase. The first column on the left presents the CMC for Simple Reaction (SR) condition related to right hand. The second column presents the CMC for SR condition related to the left hand. The third column, Complex Reaction (CR) related to right-hand. The fourth column, CR related to left hand. (A) Topographic representation of CMC values for every 64-channels. Colors were normalized using z-score for all 64-channels, red for maximum values, blue for minimum values. (B) The smoothed curves represent the CMC values for four regions of interest (ROIs) across all participants, while the dotted lines display the actual CMC values for each individual participant. The ROIs comprises Left Premotor Area (PMA), Right PMA, Left Primary Sensorimotor Area (SM1), and Right SM1. (C) Boxplots representing the CMC values of four ROIs. The dots (●) represent the participants, while the colored boxes’ height is the data range from 25th to 75th percentile, and the horizontal black line is the median value. For a better visualization, the green color of the boxes are associated with right hand, the red color with the left hand. For the SR condition, black arrows indicate the significant differences among CMC values of ROIs. *p < 0.05, **p < 0.01, ***p < 0.001.

For a better visualization of the results, we present a comprehensive summary of beta-band CMC during PREP in Fig. 4. Fig. 4 depicts in panel A, the topographic distribution on the scalp, we calculated the median values for the 64-channels across subjects to represent the distribution in one averaged scalp for each condition. In panel B, the CMC values among participants for the four ROIs; and in panel C, the differences between ROIs. As gamma-band CMC did not show significant results, we decided to present the analogue figure in Supplementary Material, as Figure S2.

### 3.3 Analysis of corticomuscular coherence (CMC) during planning (PLAN) phase

Table 4 presents the results for the beta-band CMC in each region of interest (ROI) during PLAN. Firstly, for Simple Reaction (SR) condition, the Friedman test indicated a significant effect of CMC of Left Anterior Deltoid (χ² = 13.09, *p* < 0.005), with a medium effect (*W* = 0.26). Pairwise comparisons revealed that the CMC in right PMA was significantly greater than left PMA (*Q* = 4.13, *p* < 0.05), left SM1 (*Q* = 4.51, *p* < 0.01), and right SM1 (*Q* = 3.75, *p* < 0.05). Secondly, for Complex Reaction (CR) condition, the Friedman test indicated a significant effect of CMC of Left Anterior Deltoid (χ² = 28.55, *p* < 0.0001), with a large effect (*W* = 0.56). Pairwise comparisons revealed that the CMC in right PMA was significantly greater than left PMA (*Q* = 5.82, *p* < 0.001), left SM1 (*Q* = 6.01, *p* < 0.001), and right SM1 (*Q* = 6.57, *p* < 0.001). CMC of Right Anterior Deltoid did not show significant effects in any condition. Consequently, no further pairwise comparisons were necessary for these cases.

**Table 4:** Beta-band Corticomuscular coherence (CMC) during planning phase, expressed as the median values [min – max] in arbitrary units (a.u.).

Condition	Hand to move	Left PMA (a.u.)	Right PMA (a.u.)	Left SM1 (a.u.)	Right SM1 (a.u.)	Effect size (Kendall’s W)
Simple Reaction (SR)	Right hand	0.059 [0.021 - 0.123]	0.035 [0.011 - 0.128]	0.048 [0.018 - 0.153]	0.063 [0.013 - 0.207]	NS
	Left hand	0.042 [0.015 - 0.124]	0.077* [0.02 - 0.168]	0.038 [0.004 - 0.095]	0.048 [0.012 - 0.096]	0.26
Complex Reaction (CR)	Right hand	0.096 [0.037 - 0.205]	0.096 [0.027 - 0.264]	0.084 [0.034 - 0.309]	0.112 [0.032 - 0.266]	NS
	Left hand	0.105 [0.046 - 0.295]	**0.209**⁎⁎ [0.069 - 0.341]	0.104 [0.053 - 0.276]	0.107 [0.034 - 0.191]	0.56

⁎ *p* < 0.005,.

⁎⁎ *p* < 0.0001, PMA = Premotor area, SM1 = Primary Sensorimotor area, NS = No significant.

Table 5 presents the results for the gamma-band CMC in each ROI during PLAN. For the both conditions, SR and CR, we tested CMC for the Right and Left Anterior Deltoids. The Friedman test did not show significant effects in any case. Consequently, no further pairwise comparisons were necessary for these variables.

**Table 5:** Gamma-band Corticomuscular coherence (CMC) during planning phase, expressed as the median values [min – max] in arbitrary units (a.u.).

Condition	Hand to move	Left PMA (a.u.)	Right PMA (a.u.)	Left SM1 (a.u.)	Right SM1 (a.u.)	Effect size (Kendall’s W)
Simple Reaction (SR)	Right hand	0.047 [0.007 - 0.115]	0.056 [0.018 - 0.346]	0.074 [0.016 - 0.249]	0.071 [0.005 - 0.333]	NS
	Left hand	0.052 [0.007 - 0.147]	0.042 [0.001 - 0.147]	0.037 [0.001 - 0.258]	0.052 [0.007 - 0.162]	NS
Complex Reaction (CR)	Right hand	0.07 [0.023 - 0.304]	0.077 [0.028 - 0.292]	0.064 [0.016 - 0.236]	0.078 [0.011 - 0.246]	NS
	Left hand	0.079 [0.001 - 0.292]	0.075 [0.001 - 0.579]	0.093 [0.008 - 0.257]	0.098 [0.011 - 0.286]	NS

PMA = Premotor area, SM1 = Primary Sensorimotor area, NS = No significant.

**Fig. 5:**
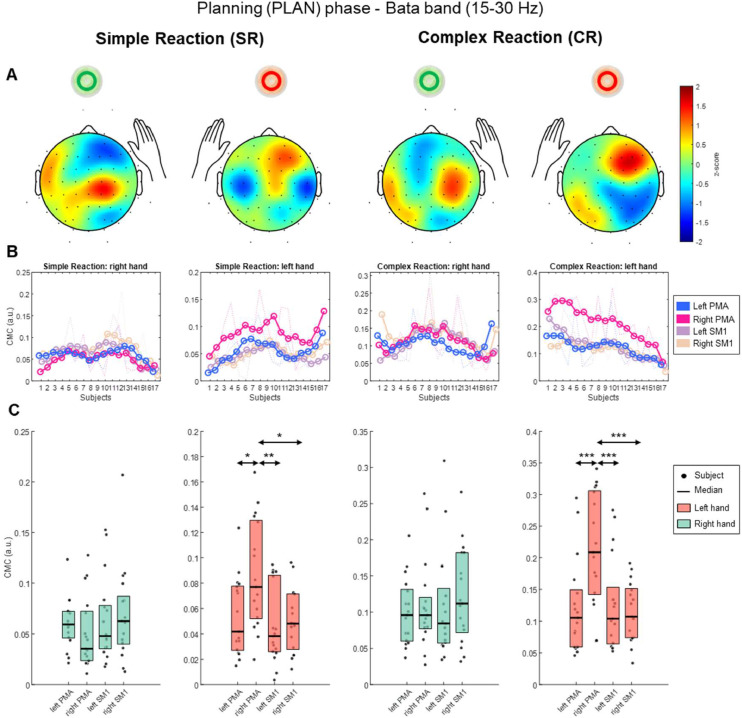
Beta-band Corticomuscular coherence (CMC) during planning phase. The first column on the left presents the CMC for Simple Reaction (SR) condition related to right hand. The second column presents the CMC for SR condition related to the left hand. The third column, Complex Reaction (CR) related to right-hand. The fourth column, CR related to left hand. (A) Topographic representation of CMC values for every 64-channels. Colors were normalized using z-score for all 64-channels, red for maximum values, blue for minimum values. (B) The smoothed curves represent the CMC values for four regions of interest (ROIs) across all participants, while the dotted lines display the actual CMC values for each individual participant. The ROIs comprises Left Premotor Area (PMA), Right PMA, Left Primary Sensorimotor Area (SM1), and Right SM1. (C) Boxplots representing the CMC values of four ROIs. The dots (●) represent the participants, while the colored boxes’ height is the data range from 25th to 75th percentile, and the horizontal black line is the median value. For a better visualization, the green color of the boxes are associated with right hand, the red color with the left hand. For the SR and CR condition during left-hand planning, black arrows indicate the significant differences among CMC values of ROIs. *p < 0.05, **p < 0.01, ***p < 0.001.

For a better visualization of the results, we present a comprehensive summary of beta-band CMC during PLAN in Fig. 5. Fig. 5 depicts in panel A, the topographic distribution on the scalp, we calculated the median values for the 64-channels across subjects to represent the distribution in one averaged scalp for each condition. In panel B, the CMC values among participants for the four ROIs; and in panel C, the differences between ROIs. As gamma-band CMC did not show significant results, we decided to present the analogue figure in Supplementary Material, as Figure S3.

## 4 Discussion

The aim of this study was to investigate the role of the premotor area during motor decision-making using functional connectivity. In line with this aim, we also explored whether pre-stimulus functional connectivity states vary across different levels of task complexity. Our findings revealed a substantial increase in beta-band corticomuscular coherence (CMC) between the agonist muscle and the contralateral premotor area (PMA), compared to other motor-related areas, prior to stimulus onset. This phenomenon was observed during preparation phase for both hands in simple reaction (SR). The same connectivity pattern was present during motor planning only for left-hand movement and remained consistent across both simple and complex reaction (CR). In contrast, we did not observe a consistent connectivity pattern for the right hand during motor planning.

### 4.1 Analysis of planning time

While previous studies have predominantly relied on Reaction Time (RT), Wong et al. (2017) have observed that several factors can modulate RT, such as context, prior experience, stimulus complexity, compatibility with the response, number of possible responses, or accuracy required. In particular, in reaching tasks, RT can be influenced by handedness. In this topic, the literature presents contradictory evidence. Dexheimer et al. (2022) and Rabbitt (1978) found shorter RTs when the task was performed with the dominant hand, whereas Velay (1999) observed shorter RTs when right-handed individuals performed the task with their left hand. Barthélémy and Boulinguez (2002) demonstrated that tasks performed with the left hand resulted in shorter RTs. Controversially, in these studies, the target was placed on the ipsilateral and/or contralateral visual field. In contrast, we placed the target in the center of the visual field. As mentioned above, these studies have used RT, thus involving electromechanical delay (EMD). EMD is suitable to be affected by the dominant hand daily use and training. Therefore, unlike them, we used planning time to reduce the neuromechanical variability. Planning time appears to be a more accurate representation of central processing, as proposed in our previous work (Cano et al., 2024b). It could represent a significant breakthrough in understanding central processes, as previously demonstrated when comparing processing speed between athletes and controls. In the present study, the results revealed that planning times were around 17 % shorter for the left hand compared to the right, despite that all participants were right handed. These differences were consistent for the both experimental conditions (i.e., SR and CR). Another consideration is that planning time for SR condition represented 50 % of the planning time for CR, consistent for the both hands. This suggests that making a decision about the arm to move is indeed a cognitive demand. Next paragraph will delve into the central processes that could elucidate these differences.

### 4.2 Motor control and brain hemisphere specialization

It is widely accepted that unilateral arm movements elicit bilateral activity in the sensorimotor areas of the cortex (Bundy and Leuthardt, 2019; Cabibel et al., 2020). The brain controls the body muscles mainly contralaterally, despite ipsilateral projections. However, the meaning of the ipsilateral cortical activation is still under discussion. For instance, Di Lazzaro et al. (1999) and Duque et al. (2005) postulated the ipsilateral hemisphere activation during limb movement as an active inhibition conducted by the contralateral sensorimotor areas for avoiding unwanted movements of the other limb. These postulates are consistent with results presented by Suffczynski et al. (2001). They proposed that movement of a body segment involves network excitation in specific cortical areas and inhibition in other areas. Regarding this topic, there are some postulates about hemisphere specializations. Johnson-Frey et al. (2005), Mancini and Mirabella (2021), and Vingerhoets (2012) showed activation on the left hemisphere (parietal and frontal regions) during movements of either the left or right hand. In contrast, the activation of the same regions in the right hemisphere was limited to movements with the left hand (contralateral). This suggests that left hemisphere perform some extra work. In the case left-hand movement has to be suppressed, the activity of both hemispheres will be disrupted. Also, the processing time required for both hemisphere activation increase, unlike one hemisphere activation, as in the case of right reaches (Mancini and Mirabella, 2021). As we mentioned above, previous studies based their conclusions about hands performance on RT measures. We used the planning time in our study and showed the opposite result; motor planning for left-hand movement required significantly less time than right hand in right-handers, consistent for both condition. This suggests that healthy participants may employ different mechanisms for motor planning depending on the hand to move.

The observed asymmetry in CMC during motor planning can be understood in the context of interhemispheric differences in motor control. Prior research has demonstrated that, in right-handed individuals, corticospinal excitability exhibits an inherent asymmetry favoring the left hemisphere, which contributes to dominant-hand responses (Poole et al., 2018). They showed when preparing a movement with the dominant right hand, corticospinal excitability remains largely unchanged in both hemispheres. In contrast, when participants are cued to prepare a movement with the non-dominant left hand, this asymmetry reverses, with an increase in right-hemisphere excitability and concurrent suppression of excitability in the left hemisphere. This hemispheric modulation during motor planning might align with our findings, in which CMC was evident between the right hemisphere and the left hand but absent in the opposite situation. The increased excitability of the right hemisphere during left-hand planning could likely enhance the sensitivity of detecting CMC. Supporting this idea, it has been demonstrated that increased corticospinal excitability induced by 20 Hz oscillatory transcranial direct current stimulation modulates beta-band CMC (Kudo et al., 2022). Conversely, the absence of significant CMC for the right hand planning suggests that left-hemisphere activity does not undergo a similar preparatory modulation, limiting the detection of coherent coupling. In line with these findings, Klein et al. (2016) reported an initial increase in corticospinal excitability between the left hemisphere and the right-hand muscle prior to muscle contraction, followed by a significant reduction, indicating greater inhibition. In their study, this phenomenon was not observed between the right hemisphere and the left-hand muscle. These findings suggest that, at least in right-handers, motor representations in the left hemisphere have a lower activation threshold than those in the right hemisphere.

Another potential explanation for the absence of detectable CMC in the right-hand motor planning is that the inhibitory function of the right frontal region might be actively engaged in a broader interhemispheric network regulating motor planning. This could disrupt or obscure the identification of beta-band CMC between the left hemisphere and the right hand, as previously suggested by Wagner et al. (2018). They showed that inhibition of right-hand movements is intrinsically linked to activation of the right-hemisphere inferior frontal cortex (rIFC). This inhibitory function is reflected in an increase in beta-band power in EEG recordings, a phenomenon particularly relevant in the context of motor actions, where rIFC activation is associated with the ability to rapidly suppress right-hand movements, which are controlled by the left hemisphere. Given the role of beta oscillations in motor planning, it is plausible that these inhibitory mechanisms contribute to the reduced CMC observed for the right dominant hand. Collectively, these findings support the notion that CMC during motor planning could be shaped by asymmetric hemispheric excitability and inhibitory processes, explaining why CMC is reliably detected for the left hand but not for the right hand in our study. This interpretation aligns with interhemispheric interaction study (Duque et al., 2007), which indicates that right-hand motor control in right-handed individuals is more distributed across both hemispheres, whereas left-hand control relies more heavily on the right hemisphere. To elucidate whether these interhemispheric differences are consistent across individuals, future assessments on left-handed participants would provide a more comprehensive characterization of hemispheric similarities and differences in motor planning. Our bivariate approach allows us to examine the functional connectivity between the cortex and muscles, going beyond EEG activity in the time or frequency domain. However, to further investigate regional interactions and identify inhibitory networks, additional analytical approaches should be employed.

### 4.3 Motor planning and premotor area

It is well known that PMA is involved in a variety of motor actions (Graziano and Aflalo, 2007). Pilacinski and Lindner (2019) demonstrated the relevance of PMA in the hand trajectories using fMRI and tested PMA activation for non-straight movement planning. Our findings agree with previous work (Lemon, 2008) pointing out the PMA as an early-motor-organization area, also in line with previous studies that showed the activity -not connectivity- of the left hemisphere PMA in right-handers during movement preparation (Hammond and Fox, 2005) and action selection (Schluter et al., 2001). Our findings revealed functional connectivity in PMA modulated in beta band. This signature was observed in the PMA contralateral to the agonist muscle before visual stimulus presentation –during preparation phase– only in the SR condition. It may be interpreted as an anticipatory state preparing for the expected stimulus, as participants already knew which hand should be moved. This phenomenon was not observed in the CR condition as expected. In this line, Chandrasekaran et al. (2019) observed beta-band activity before and after of the visual stimulus onset but not during the arm movement on rhesus macaques. Additionally, Bruijn et al. (2015) suggested that the PMA may be more related to intention rather than muscle activation.

As demonstrated by Pfurtscheller and Lopes da Silva (1999) and subsequent studies on event-related synchronization/desynchronization (ERS/ERD), brain oscillations, particularly in the beta band, play a crucial role in motor command generation. CMC can be detected in several brain regions (Yang et al., 2016), also during passive states (Porcaro et al., 2018). Our study aimed to investigate whether CMC differs between regions of interest just before muscle contraction. We observed a significant increase in beta-band CMC in the contralateral PMA during the preparation phase, suggesting that the PMA is actively involved in motor preparation. This indicates that the brain is establishing a neural connection with the muscles in anticipation of the task, despite no overt muscle activity. During motor planning, the brain engages in cognitive processes such as motor response selection. The observed modulation of CMC reflects the brain’s effort to optimize motor commands for required responses. Thus, the changes in CMC during motor planning underscore the brain's preparatory mechanisms prior to muscle activity, i.e. motor command encoding.

On the other hand, several studies on humans combining EEG and transcranial magnetic stimulation (TMS) (Bruijn et al., 2015; Busan et al., 2012; Duque et al., 2005; Zrenner et al., 2022) support the idea of PMA is involved in active planning, but also in inhibitory. For instance, Duque et al. (2005) suggested that PMA plays a critical role for motor plan selection, especially in the context of visuomotor associations. They applied TMS over PMA in hand selection after a cue, and showed that PMA not only involved in movement selection but also generate inhibitory signals for blocking motor output until the movement is initiated. Wagner et al. (2018) revealed a beta-band signature in the EEG in the right frontal lobe serves as a temporally precise neural marker for the response inhibition process. Interestingly, in our study, after the stimulus presentation –during planning phase–, significant functional connectivity was observed in PMA contralateral to the agonist muscle only for the left-hand motor planning, consistent for the both SR and CR condition. These findings suggest asymmetrical motor planning between the hands in healthy participants. Specifically, the evidence indicates that the left PMA does not play the same role as the right PMA, at least in connectivity with the contralateral agonist muscle. Consequently, motor planning for each hand is not analogous across hemispheres, consistent with our previous work (Cano et al., 2024a). Moreover, since the PMA may play a role in motor inhibition, future studies should focus on investigating functional connectivity between brain regions.

### 4.4 Beta and Gamma oscillations

We did not observe any connectivity patterns in the gamma band during the preparation or planning phases. However, this does not imply that gamma-band activity is unrelated to sensorimotor functions. For instance, Mehrkanoon et al. (2014) suggested that CMC in distinct frequency bands facilitates different modes of neural communication between the motor cortex and the spinal cord. While beta-band CMC may reflect the maintenance of the current state (also named ‘status quo’), alpha- and gamma-band coherence appear to highlight the feedback and feedforward interactions involved in detecting prediction errors associated with movement overshoot. In this line, Chakarov et al. (2009) postulated different functional roles of CMC in the two frequency ranges: the increase of force level is associated with larger beta-band CMC while the gamma CMC allows rapid sensorimotor and cognitive integration during dynamic force output. The lack of gamma-band CMC signature in our study could be related to the short time interval selected to be analyzed. As Kenville et al. (2020) noted from a mechanistic perspective, gamma-band oscillations are thought to reflect network integration, while beta-band oscillations are primarily associated with corticomotor drive. Beta-band CMC is most prominent during tonic muscle contractions and disappears during movement (Mehrkanoon et al., 2014). While there is still no consensus, beta-band oscillations seem to play an executive, initiating role during motor actions, whereas gamma oscillations are attributed a sensory integrative function. In agreement with such notion, our results align with the previous expectations regarding the role of beta-band CMC in motor planning, rather than that of gamma.

The present study reveals that focusing on CMC during motor planning provides an innovative approach for assessing brain dynamics of motor control. This methodology could be employed to evaluate mental states and cognitive workload in real time, especially when combined with emerging wearable technologies as suggested by Chang et al. (2023). It also holds potential for tracking progress during therapy or physical training programs by examining both the temporal aspects and functional connectivity involved in motor planning. For instance, studies in hybrid brain-computer interfaces suggest that EEG-EMG connectivity patterns offer more reliable control features than traditional event-related potential-based algorithms (De Seta et al., 2022). In rehabilitation contexts, CMC networks have been shown to capture motor abnormalities effectively, supporting the idea that combining CMC with intermuscular coherence could lead to more effective rehabilitation strategies (Colamarino et al., 2021; Pichiorri et al., 2023). Furthermore, in the field of prosthetic control, research suggests that CMC-based systems could significantly improve the performance and clinical applicability of brain-robot interfaces (Chowdhury et al., 2019). Our findings align with these developments and open pathways for using CMC in motor control, offering insights into early movement intention detection, with potential applications in therapeutic and assistive technologies. By incorporating motor tasks with incremental complexity, our approach may facilitate the assessment of changes in brain connectivity associated with motor learning processes and neuroplasticity in healthy individuals (Beck et al., 2021). Additionally, CMC has been suggested to reveal differences in motor control adaptations among individuals with neuromuscular impairments, such as stroke (Aikio et al., 2021; Fauvet et al., 2021; Von Carlowitz-Ghori et al., 2014) or spinal cord injuries (Cremoux et al., 2017). In this context, our proposal could contribute to understanding the mechanisms underlying alterations in sensorimotor processing.

It is important to consider some potential limitations of our study. The relatively small sample size of seventeen participants, although sufficient to detect the primary effects of interest, may limit the generalizability of the findings, despite adhering to the sample estimation outlined in the a-priori power analysis with *ß* > 0.8. It is also important to note that the effect sizes for the repeated measures statistical analysis were predominantly large (>0.5) or medium (0.2–0.5). A larger sample would enhance statistical power and allow for a more comprehensive exploration of inter-individual variability. Additionally, the exclusive inclusion of right-handed participants restricts the ability to generalize the findings to left-handed individuals, who may exhibit different patterns of motor cortical activity and connectivity (Gutwinski et al., 2011). Caution should be exercised to interpret the interhemispheric differences in PMA. Future studies should aim to include a balanced sample with both left- and right-handed participants to examine potential differences in cortical dynamics and to ensure broader applicability of the results. These considerations highlight the need for caution when interpreting the findings and underscore the importance of extending this line of research to address these limitations.

Our study employed a fixed window to analyze the preparation phase within a defined time range, ensuring standardization across conditions and minimizing potential contamination from movement artifacts and anticipatory mechanisms that could otherwise confound the results. This approach effectively revealed spatial patterns of CMC distribution in the contralateral PMA during the simple reaction task. Conversely, the absence of these expected patterns in the uncertainty condition strongly supports the idea that, during the preparation phase of complex reactions, movement was not pre-planned. This methodological choice ensures that the subsequent analysis of the motor planning phase remains unbiased by residual preliminary connectivity. Future studies could explore an alternative approach using staged time windows for the preparation phase, guided by cortical signal fluctuations. This strategy may provide a more detailed characterization of preparatory activity by capturing the dynamic evolution of connectivity states leading up to stimulus presentation. Such an approach could help identify distinct neural states during the waiting and preparation phases, offering further insights into individual differences in motor preparation strategies and their relationship with task complexity.

Finally, handedness was assessed by asking participants which hand they used for writing, as this served as a practical indicator of the arm with greater daily usage. However, specific evaluations of dominance were not conducted. Although formal assessments like the Edinburgh Handedness Inventory (EHI) are commonly used to evaluate hand dominance, research has shown that self-reported handedness is also a reliable measure in research settings. For instance, Ransil and Schachter (1994) demonstrated that self-reported global handedness (left, right, ambidextrous) showed high test-retest reliability over an 18-month period, comparable to that of the EHI. In addition, Ransil and Schachter (1998), in a study with 1196 professionals grouped in nine professions, showed similar results. Coren (1993) explored three self-report measures of handedness and found that both brief (4-item) and extended (12-item) inventories produced 98.8 % classification concordance in a sample of 250 subjects. More recently, Leppanen et al. (2019) investigated subjects who completed a modified EHI, in which higher scores indicated more consistent usage of the preferred hand, observing that subjects were faster when using their preferred hand, and the magnitude of the preferred hand advantage was positively correlated with self-reported consistency.

## 5 Conclusions

This study provides evidence for the role of the premotor area in the motor planning of hand movements. We found significant functional connectivity in the beta band between the left Anterior Deltoid muscle and the right premotor area, compared to other motor-related areas. This connectivity pattern was consistent across two experimental conditions involving motor decision-making, which included both simple and complex reaction tasks. In contrast, right-hand motor planning did not exhibit the same pattern, possibly indicating more complex brain process compared to the left hand in right-handed individuals.

Our findings align with previous research, supporting the notion that the premotor area plays a critical role in motor decision-making, though not symmetrically across hemispheres. The theoretical implications of these findings are relevant for understanding the complex relationship between the cortex and muscles during motor decision-making. This interaction is essential for uncovering the central nervous system's strategies in planning motor actions, advancing our understanding of the underlying mechanisms of motor control. On the practical side, this study offers a novel approach to exploring motor planning through functional connectivity within short time intervals, around 200 milliseconds.

## Ethics statement

All procedures performed in studies involving human participants were in accordance with the ethical standards of the institutional and national research committee and with the 1964 Helsinki Declaration and its later amendments or comparable ethical standards. The Bioethics Committee of the Miguel Hernandez University from Elche, Spain approved the study (Reference: IB.EFJ.04.21). Informed consent was obtained from all individual participants included in the study.

## CRediT authorship contribution statement

**Leonardo A. Cano:** Conceptualization, Methodology, Investigation, Data curation, Formal analysis, Software, Writing – original draft, Writing – review & editing. **Ana L. Albarracín:** Conceptualization, Methodology, Investigation, Formal analysis, Writing – review & editing. **Fernando D. Farfán:** Conceptualization, Methodology, Investigation, Data curation, Formal analysis, Software, Writing – review & editing, Funding acquisition, Project administration, Resources, Supervision. **Eduardo Fernández:** Methodology, Investigation, Writing – review & editing, Funding acquisition, Project administration, Resources, Supervision.

## Declaration of competing interest

The authors declare no competing interests.

## Data Availability

Data will be made available on request.

## Appendix Supplementary materials

Image, application 1
