# Supplementary material for: Brain-hemispheric differences in the premotor area for motor planning: An approach based on corticomuscular connectivity during motor decision-making
Source: Neuroimage. 2025 May 15;312:121230. doi: 10.1016/j.neuroimage.2025.121230 (PMC12055607; doi:10.1016/j.neuroimage.2025.121230)
Supplement: Supplementary file 1 [file mmc1.docx]

**SUPPLEMENTARY MATERIAL**

**Section 2.7. Procedure for computing signal coupling**

The procedure we applied to compute Corticomuscular Coherence (CMC) is based on Bigot et al^1^ method in a similar way to its recent applications ^2–7^. Here, we present a step-by-step explanation with an example between two signals.

**1.** Select two signals: EEG channel (FC3 electrode) and EMG channel (right Anterior Deltoid).

**2.** Select repetitions to include: repetitions with duration within the range of the mean ± 2 SD.

**3.** Apply Continuous Wavelet transform of both segmented signals, for each repetition. In this step, WavCrossSpect toolbox by Bigot et al ^1^ was used with the parameters: 'Mother'=Morlet, 'nvoice'=5, 'J1'=100, 'wavenumber'=6, 'MaxScale'=default). This step provides an Auto-Spectrum (scalogram or time-frequency map) for every signal segment, for both EEG and EMG.

**4.** Compute 'Cross-Spectrum' between signals pair, for each repetition separately. This is a cross correlation between the two (EEG and EMG) auto-spectrums obtained on step 3.

**5.** Compute ‘Mean Auto-Spectrum’ for each signal separately (Fig. S1 A and B), which is calculated as the point-by-point mean from the all power auto-spectrums of repetitions selected on step 3.

**6.** Compute ‘Mean Cross-Spectrum’ using ‘Cross-Spectrum’ from each signals pair (Fig. S1 C), which is calculated as the point-by-point mean from every cross-spectrums computed on step 4.

**7.** Determine ‘Significant Cross-Spectrum’ (Fig. S1 E), which is the points from ‘Mean Cross-Spectrum’ map that are above the threshold $\lambda_{\alpha}$ obtained with Equation S1, at level $\alpha=0.05$, explained in detail by Bigot et al ^1^.

| $\lambda_{\alpha}=\frac{\rho_{x} \rho_{y}}{n} \left( -log \left( \alpha/2 \right)+\sqrt{-2n\log\left( \alpha/2 \right)} \right) ,$ | (Eq. S1) |
| --- | --- |

where $\rho_{x}$ and $\rho_{y}$ are the largest eigenvalues of the empirical covariance matrices of both signals; and $n$ is the number of repetitions.

**8.** Compute ‘Magnitude-Squared Coherence’ $R_{xy}^{2}\left( \omega,u \right)$, which is a normalized value from 0 to 1 (Fig. S1 D), with Equation S2.

| $R_{xy}^{2}\left( \omega,u \right)=\frac{{{\vert S}_{xy}\left( \omega,u \right)\vert}^{2}}{S_{x}\left( \omega,u \right) S_{y}\left( \omega,u \right)} ,$ | (Eq. S2) |
| --- | --- |

where $S_{xy}\left( \omega,u \right)$ is the ‘Mean Cross-Spectrum’; $S_{x}\left( \omega,u \right)$ and $S_{y}\left( \omega,u \right)$ are the ‘Mean Auto-Spectrums’ of both signals.

**9.** Apply 'Significant Cross-Spectrum' matrix as a mask over 'Magnitude-Squared Coherence' to obtain normalized coherence only where the ‘Mean cross-spectrum’ was significant (Fig. S1 F).

**10.** Boundary selection for the windows of interest within the time-frequency map depending of frequency and time interval. We decided to analyze the beta (15 to 30 Hz) and gamma band (30 to 45 Hz). The windows of interest are marked with dashed lines in Fig. S1 G.

**11.** Compute CMC as the single value (mean) of all values within each window of interest.

**12.** The same procedure was performed between every EEG channel and the corresponding EMG channel.


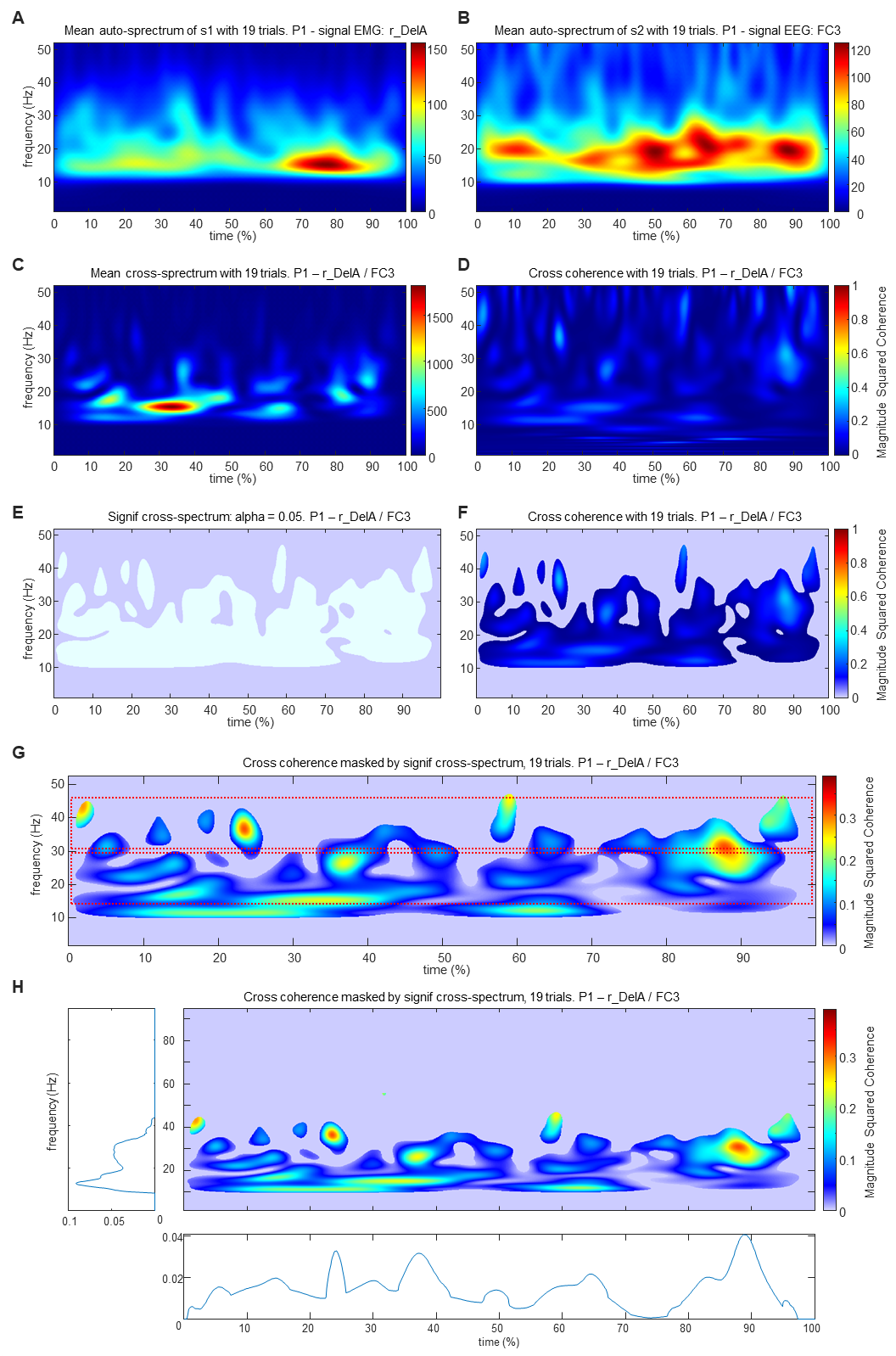


**Figure S1.** Summary for CMC compute procedure. (A) Compute 'Mean Auto-Spectrum' from the 'Wavelet Scalogram' of n repetitions for signal 1 (e.g. right Anterior Deltoid). (B) Compute 'Mean Auto-Spectrum' from the 'Wavelet Scalogram' of n repetitions for signal 2 (e.g. FC3 EEG-channel). (C) Compute 'Mean Cross-Spectrum' from the 'Cross-Spectrums' from each signals pair. (D) Compute 'Magnitude-Squared Coherence'. (E) Determine 'Significant Cross-Spectrum'. (F) Use 'Significant Cross-Spectrum' as a mask over 'Magnitude-Squared Coherence' (G) Coherence masked with color scale adjusted. The dashed rectangles in red correspond to the windows of interest to compute CMC values, for beta-band (15 to 30 Hz) and gamma-band (30 to 45 Hz). (H) On the center, the resultant time-frequency map. On the left, a graph showing the mean CMC curve as a function of frequency. On the bottom, graph showing the mean CMC curve as a function of time.

**Section 3.2. Summary of results for gamma-band CMC during preparation phase**


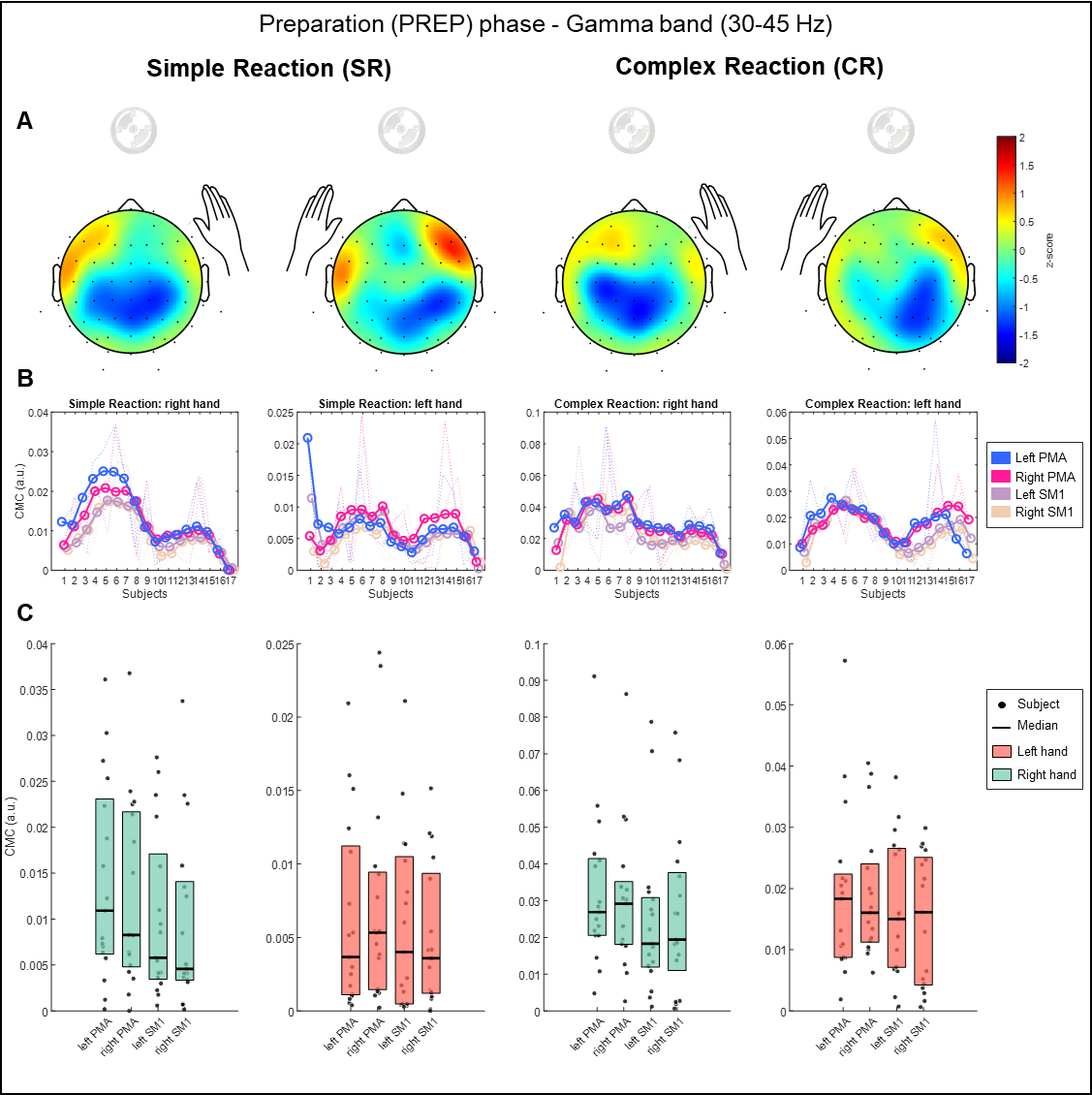


**Figure S2.** Gamma-band Corticomuscular coherence (CMC) during preparation (PREP) phase. The first column on the left presents the CMC for Simple Reaction (SR) condition related to right hand. The second column presents the CMC for SR condition related to the left hand. The third column, Complex Reaction (CR) related to right-hand. The fourth column, CR related to left hand. (A) Topographic representation of CMC values for every 64-channels. Colors were normalized using z-score for all 64-channels, red for maximum values, blue for minimum values. (B) The smoothed curves represent the CMC values for four regions of interest (ROIs) across all participants, while the dotted lines display the actual CMC values for each individual participant. The ROIs comprises Left Premotor Area (PMA), Right PMA, Left Primary Sensorimotor Area (SM1), and Right SM1. (C) Boxplots representing the CMC values of four ROIs. The dots (●) represent the participants, while the colored boxes’ height is the data range from 25^th^ to 75^th^ percentile, and the horizontal black line is the median value. For a better visualization, the green color of the boxes are associated with right hand, the red color with the left hand.

**Section 3.3. Summary of results for gamma-band CMC during planning phase**


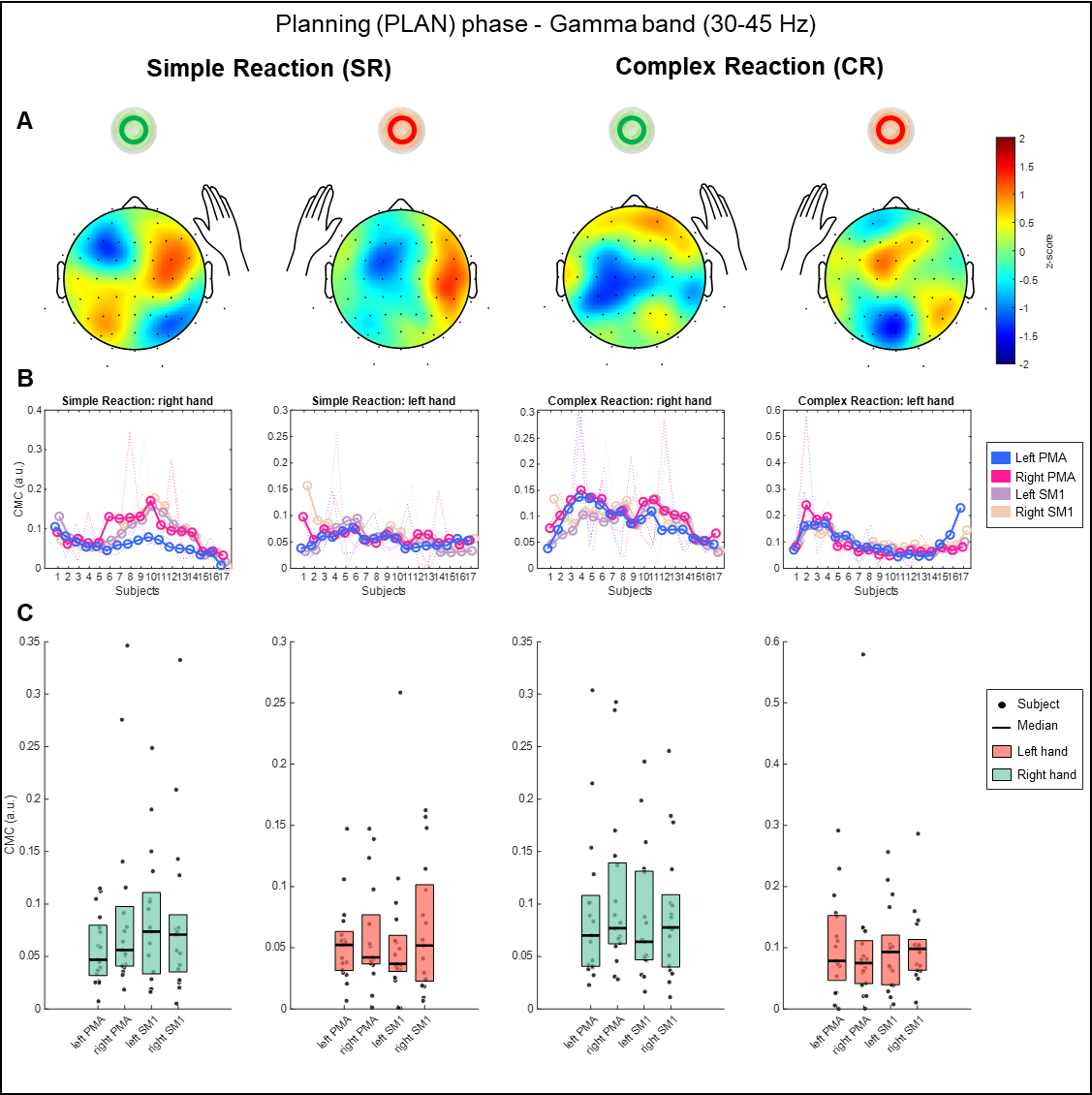


**Figure S3.** Gamma-band Corticomuscular coherence (CMC) during planning phase. The first column on the left presents the CMC for Simple Reaction (SR) condition related to right hand. The second column presents the CMC for SR condition related to the left hand. The third column, Complex Reaction (CR) related to right-hand. The fourth column, CR related to left hand. (A) Topographic representation of CMC values for every 64-channels. Colors were normalized using z-score for all 64-channels, red for maximum values, blue for minimum values. (B) The smoothed curves represent the CMC values for four regions of interest (ROIs) across all participants, while the dotted lines display the actual CMC values for each individual participant. The ROIs comprises Left Premotor Area (PMA), Right PMA, Left Primary Sensorimotor Area (SM1), and Right SM1. (C) Boxplots representing the CMC values of four ROIs. The dots (●) represent the participants, while the colored boxes’ height is the data range from 25^th^ to 75^th^ percentile, and the horizontal black line is the median value. For a better visualization, the green color of the boxes are associated with right hand, the red color with the left hand.

**SUPPLEMENTARY REFERENCES**

1. Bigot, J., Longcamp, M., Dal Maso, F., and Amarantini, D. (2011). A new statistical test based on the wavelet cross-spectrum to detect time–frequency dependence between non-stationary signals: Application to the analysis of cortico-muscular interactions. NeuroImage *55*, 1504–1518. https://doi.org/10.1016/j.neuroimage.2011.01.033.

2. Tisseyre, J., Cremoux, S., Amarantini, D., and Tallet, J. (2022). Increased intensity of unintended mirror muscle contractions after cervical spinal cord injury is associated with changes in interhemispheric and corticomuscular coherences. Behav. Brain Res. *417*, 113563. https://doi.org/10.1016/j.bbr.2021.113563.

3. Fauvet, M., Gasq, D., Chalard, A., Tisseyre, J., and Amarantini, D. (2021). Temporal Dynamics of Corticomuscular Coherence Reflects Alteration of the Central Mechanisms of Neural Motor Control in Post-Stroke Patients. Front. Hum. Neurosci. *15*, 682080. https://doi.org/10.3389/fnhum.2021.682080.

4. Glories, D., Soulhol, M., Amarantini, D., and Duclay, J. (2021). Specific modulation of corticomuscular coherence during submaximal voluntary isometric, shortening and lengthening contractions. Sci. Rep. *11*, 6322. https://doi.org/10.1038/s41598-021-85851-w.

5. Elie, D., Barbier, F., Ido, G., and Cremoux, S. (2021). Corticomuscular Coherence and Motor Control Adaptations after Isometric Maximal Strength Training. Brain Sci. *11*, 254. https://doi.org/10.3390/brainsci11020254.

6. Desmyttere, G., Mathieu, E., Begon, M., Simoneau‐Buessinger, E., and Cremoux, S. (2018). Effect of the phase of force production on corticomuscular coherence with agonist and antagonist muscles. Eur. J. Neurosci. *48*, 3288–3298. https://doi.org/10.1111/ejn.14126.

7. Dal Maso, F., Longcamp, M., Cremoux, S., and Amarantini, D. (2017). Effect of training status on beta-range corticomuscular coherence in agonist vs. antagonist muscles during isometric knee contractions. Exp. Brain Res. *235*, 3023–3031. https://doi.org/10.1007/s00221-017-5035-z.
